# Genetic and epigenetic variations in BDNF gene involved in Anorexia Nervosa

**DOI:** 10.1192/j.eurpsy.2022.1743

**Published:** 2022-09-01

**Authors:** N. Ramoz, G. Maussion, J. Clarke, P. Gorwood

**Affiliations:** 1 University of Paris, Ipnp, Inserm U1266, Paris, France; 2 McGill University, The Neuro’s Early Drug Discovery Unit (eddu), Montreal, Canada; 3 GHU Psychiatry & Neurosciences Paris, Clinique Des Maladies Mentales Et De L’encéphale (cmme), Centre Hospitalier Sainte-anne, Paris, France

**Keywords:** DNA methylation, Neuropsychiatry, biomarker, Dosage

## Abstract

**Introduction:**

Anorexia nervosa (AN) is a chronic psychiatric disorder resulting from abnormal eating habits with a high prevalence (0.5%). AN involves genetic and epigenetic factors supporting that AN is a metabo-psychiatric disorder. One candidate gene for AN, validated by meta-analyzes, is *BDNF* which encodes the brain-derived neurotrophic factor. BDNF negatively modulates the central control of food intake and its injection in rodents induces weight loss and anorexia. In humans, we observed an association of its functional variant Val66Met/rs6265 and electrodermal response to images of thinness suggesting an association between rs6265 and a reward effect of weight loss in AN.

**Objectives:**

This work study the impact of the functional polymorphism at risk rs6265, epigenetic variations in DNA methylation of *BDNF* gene and consequences on the concentrations of BDNF in AN patients.

**Methods:**

DNA was isolated from 24 AN patients and 48 controls. DNA methylation was measured for sites spanning the BDNF gene using Infinium HumanMethylation450 BeadChip technology. The genotyping of rs6265 was performed by Taqman-SNP assay. The BDNF was dosaged by ELISA from plasmas.

**Results:**

We observe that several sites are significantly hypermethylated in AN patients compared to controls. AN patients show significantly higher BDNF levels than controls. Finally, this BDNF concentration is significantly higher in AN carrying the risk Met66 allele.

**Conclusions:**

This work demonstrates the effects of genetic and epigenetic variations of *BDNF*, which could constitute relevant diagnostic biomarkers of AN, and their likely consequences in the pathophysiology of AN. *This work was supported by the Nestlé Foundation.*

**Disclosure:**

No significant relationships.

